# Health and Environmental Impacts of Major Foods Consumed in Regional Food Systems of Brazil

**DOI:** 10.3390/ijerph22050745

**Published:** 2025-05-09

**Authors:** Marhya Júlia Silva Leite, Lucas de Almeida Moura, Eduardo De Carli, Dirce Maria Lobo Marchioni, Olivier Jolliet, Eliseu Verly, Aline Martins de Carvalho

**Affiliations:** 1 Department of Nutrition, Faculty of Public Health, University of São Paulo (USP), São Paulo 01246-904, Brazil; marhyajulialeite@usp.br (M.J.S.L.); lucasdemoura@usp.br (L.d.A.M.); edecarli@usp.br (E.D.C.); marchioni@usp.br (D.M.L.M.); 2 Department of Environmental and Resource Engineering, Technical University of Denmark (DTU), Copenhagen 2800, Denmark; ojoll@dtu.dk; 3 Department of Epidemiology, Institute of Social Medicine, State University of Rio de Janeiro (UERJ), Rio de Janeiro 20550-900, Brazil; eliseujunior@gmail.com

**Keywords:** health impact, environmental impact, disability-adjusted life years, ultra-processed foods, Brazil, food systems, sustainability

## Abstract

This study examines the relationship between the 1141 most consumed foods in Brazil and their individual and combined health and environmental impacts. Foods are analyzed across different food system clusters, based on the health burden (DALYs) in minutes of healthy life using the Health Nutritional Index (HENI), greenhouse gas emissions, and water use. The most consumed foods were in natura products, such as rice, beans and meat, and a few ultra-processed products such as biscuits and soft drinks. Our results revealed an average HENI of −5.89 min, with values varying from −39.69 min of healthy life (stuffed cookies) to 17.22 min (freshwater fish). Animal-derived products, particularly red meat, had the highest environmental costs, contributing significantly to greenhouse gas emissions and water use. In contrast, plant-based foods like beans and fruits had better HENI scores and lower environmental impacts. We also found that greenhouse gas emissions reached up to 21.3 kg CO_2eq_ (beef dish), and water use peaked at 306.1 L (mozzarella pizza). Our findings provide valuable insights into the real-world consequences of individual and institutional food choices, demonstrating their measurable impacts on health and the environment. By moving beyond theoretical assumptions, this evidence strengthens the case for integrating sustainability into public food policies, including dietary guidelines that consider regional specificities and environmental concerns alongside nutritional recommendations.

## 1. Introduction

Currently, climate change, obesity, and undernutrition characterize a global syndemic representings one of the greatest challenges of the modern era as it threatens global food and nutritional security [1]. This scenario highlights the contradiction between healthy, sustainable agricultural systems and the expansion of agribusiness and large food industries, showing that food systems are one of the main drivers of the global syndemic. Using the High Level Panel of Experts (2017), a food system framework, food systems can be understood as comprising four interconnected components: food production, food environments, food consumption, and their impacts on both health and the environment [2].

In terms of food production, in 2022, Brazil emitted a total of 2.3 million tons of CO_2_ equivalent, with cattle production responsible for 77.6% of the greenhouse gases from the food system, making the country one of the largest global emitters, especially in the Legal Amazon, where deforestation is driven by livestock farming [3,4]. Since 2019, deforestation in the Amazon has exceeded 10,000 km^2^ annually, further intensifying environmental concerns related to land use and emissions. Data from 1985 to 2019 indicate a total deforested area of approximately 10.2% of the Amazon, highlighting the significant land conversion over the decades and its environmental implications [3,4].

Regarding the consumption part of the Brazilian food system, beef is highly consumed and accounts for 86% of the carbon footprint of the diet, in addition to being responsible for 90% of land use, 77% of eutrophication, and 26% of water use associated with food production in 2017 [5]. Agribusiness alone uses about 71% of the country’s water resources [6], with beef being the food with the greatest environmental impact, both in terms of Greenhouse Gas (GHG) emissions, including carbon dioxide (CO_2_) and methane (CH_4_), and water use [7].

Furthermore, studies on the Brazilian diet show that, despite the high consumption of unprocessed foods, there is an increase in the consumption of ultra-processed foods, which represent 20% of the calories consumed by the Brazilian population [8,9,10,11]. This trend has significant health implications, as noncommunicable diseases (NCDs) are the leading cause of death worldwide, accounting for 74% of global deaths in 2019 [12]. In Brazil, 52% of Brazilians have at least one NCD, with 14% of deaths and 8% of Disability-Adjusted Life Years (DALYs) attributed to dietary factors, according to the Global Burden of Disease (2019) [12]. Additionally, ultra-processed foods were associated with 11% of premature deaths in adults aged 30 to 69 in the same year [13]. These statistics highlight the significant role of diet in shaping health outcomes and underline the urgent need for strategies to promote healthier eating habits.

While considerable effort has been made to analyze the health and environmental impacts of diets, few studies have integrated these dimensions into comprehensive food indices, particularly in regions like the Global South [14]. In Latin America, and specifically in Brazil, there is still a significant gap in the use of quantitative metrics to assess, in particular, the environmental impacts of diets. Most existing indices, such as the Health Nutritional Index (HENI) [15], have been developed primarily in high-income countries and may not adequately reflect the specificities of Brazilian food systems and consumption patterns [16,17].

Addressing this gap, the present study seeks to adapt, for the first time, a nutritional index based on the most consumed foods in the four major regional food systems in Brazil. By applying the HENI methodology in this context and incorporating both health and environmental metrics, our research provides a novel perspective, offering a more representative assessment of the impacts of dietary choices in the country. This approach links diet to health and environmental effects. Given the poor quality of the Brazilian diet [18], along with its well-documented negative implications for both health and the environment, there is an urgent need for tools that can support the development of more sustainable food policies. Our study aims to examine the relationship between the most consumed foods in different parts of Brazil and their individual and combined health and environmental impacts. Ultimately, this research seeks to foster a gradual transition toward diets that enhance both individual well-being and environmental sustainability.

## 2. Materials and Methods

For the development of this study, the Health Nutrition Index (HENI) impact was adapted, using estimates of the dietary environmental impact, including greenhouse gas emissions (kg of CO_2_ equivalents) and water use (L), based on the methodology proposed by Stylianou et al. (2021) [15]. This approach combines nutritional and environmental indicators to identify foods that are environmentally sustainable and promote health [15].

To calculate the HENI for this study, the net beneficial or harmful health burden was determined in terms of healthy life minutes linked to the average portion size of the most consumed foods in Brazil, considering the demographics and health conditions of the Brazilian population. The research utilized data from the food consumption database of the Brazilian population derived from the Household Budget Survey—National Food Survey (INA 2017–2018) [16], the Nova food processing classification [19], the classification of Brazilian regional food systems identified by the Brazilian Multidimensional Index for Sustainable Food Systems (MISFS-R) [20], and environmental parameters related to water use and greenhouse gas emissions from food production in Brazil [21].

### 2.1. Brazilian Health Nutritional Index Estimation

The index utilizes parameters from the Global Burden of Disease (GBD) [12], which correlates dietary choices with disease mortality, based on 15 dietary risk factors: fruits; vegetables; legumes; whole grains; nuts and seeds; fiber; calcium; omega-3 fatty acids; polyunsaturated fatty acids; and milk, in addition to high levels of red meat; processed meat; sodium; trans fats; and sugar-sweetened beverages.

Foods receive scores that indicate their health impact: positive scores represent foods that contribute to increased healthy life minutes, while negative scores are associated with potential long-term health risks.

#### 2.1.1. Calculation Procedure

The calculation and reasoning were first presented by Stylianou et al. (2021) based on the 2019 GBD framework. Dietary Risk Factors (DRFs) were assessed using a comparative risk evaluation approach tailored for marginal adjustments in food consumption (exposure) related to the addition or removal of specific food items from the diet [15,22]. The process of calculating DRFs included several steps: establishing dose–response relationships, determining baseline exposure levels, and assessing dietary risk factors. This method was specific to sex and age groups to more accurately estimate an individual’s exposure to dietary risks, recognizing that each person has a distinct dietary pattern.

##### Determining Baseline Exposure Levels

Age–sex-specific average exposure levels for 15 dietary risks were derived from the 2017–2018 National Food Survey (INA 2017–2018) [16]. The survey comprises 46,164 individuals over the age of 10 across all regions of the country. Dietary consumption was collected through 2 non-consecutive 24 h recalls.

##### Calculating Dietary Risk Factors

The DRFs proposed by Stylianou et al. (2021) [15] were modified to account for the non-linearity in the dose–response relationship for relative risks (*RRs*). The *DRFs* for each dietary component were calculated according to the Equation (1):

(1) $$DRF=\frac{{RR}_{x}-1}{x}\cdot \frac{\mu BR}{\sum \;P_{x}{RR}_{x}}$$

where *x* represents the exposure level; *RR* is the relative risk at exposure level *x*; *P* indicates the population at exposure level *x*; and *μBR* is the burden rate converted to microDALYs (*μBR* = (*BR*/100,000/365.25) × 10^6^). Here, *BR* stands for the burden rate (per 100,000 individuals). This equation was applied to each age-sex group in Brazil for every diet-disease pair. The overall DRF was computed as the average of the age–sex-specific DRF, weighted by population size.

For sodium, DRFs encompass both direct health effects (such as stomach cancer) and indirect effects mediated by elevated systolic blood pressure (SBP), with age, race, and hypertension status acting as effect modifiers (Equation (1)).

##### Disease Burden

The Health Nutritional Index (HENI) for a food item is a recalculated measure of the total Disability-Adjusted Life Years (DALYs) associated with dietary risk components, expressed as DALYs per gram of those components [15]. DALYs quantify the sum of years lost due to premature mortality, reduced health, or disability caused by exposure to risk factors, such as dietary risks. These dietary risks include calcium, fiber, seafood-derived omega-3 fatty acids, polyunsaturated fats, trans fats, sodium, fruits, vegetables, milk, legumes, nuts and seeds, red meat, processed meat, and whole grains. Data on DALYs related to insufficient or excessive intake levels of these components were sourced from the Global Burden of Disease (GBD) study [12].

The HENI measures the minutes of healthy life gained or lost due to a marginal change in the dietary risk component content of an adult’s diet, under the assumption that the effects of multiple dietary risks are independent and additive and that components not addressed by the GBD study have neutral effects on health. This methodology was adapted from Stylianou et al. (2021) [15], incorporating updated background data, including the 2019 GBD relative risks, and applying burden rates specific to the Brazilian population instead of U.S. rates.

Calculating HENI scores involved three main steps: (1) estimating the Dietary Risk Factors (DRFs) for the Brazilian population using disease-specific burden rates; (2) determining the risk factor content of individual food items based on the GBD (2019) [12] database; and (3) multiplying the DRFs by the risk factor content to compute the HENI for each food item. DRFs were derived using nonlinear optimization to establish optimal dose–response curves based on 81 risk–outcome-specific relative risks from the GBD. This allowed for the calculation of the change in disease risk per gram of dietary risk component intake. These results were combined with the burden rates (expressed as μDALYs per 100,000 people per year) for each corresponding disease in Brazil to generate DRFs, reflecting the μDALYs lost or gained per gram of dietary risk component intake.

Each dietary risk factor (DRF) was multiplied by the amount of the respective risk component (in grams) present in the average portion size of the analyzed food (for example, the sodium content in an average portion of rice). Then, the risks were aggregated, and the net estimate was converted from μDALYs to minutes of healthy life using the conversion formula (1μDALY = 1 year of healthy life lost × 365 days × 24 h × 60 min × 10^−6^ = −0.53 min of healthy life), resulting in the food-specific HENI score (Figure S1 in the Supplementary Materials).

### 2.2. Most Consumed Foods

#### Food and Nutrient Database

A total of 2534 foods were referenced in the 2017–2018 National Food Survey (INA), including synonyms and different preparation methods for 1279 unique foods/drinks. After grouping synonyms and consolidating variations, the list used based on these secondary data was refined to a total of 1141 distinct foods and beverages. To align the composition of these foods with the definitions and measurement units of the nine food groups (fruits, vegetables, legumes, whole grains, seeds, milk, red meat, processed meat, and sugar-sweetened beverages) and six nutrients (fiber, calcium, fish omega-3, polyunsaturated fatty acids—omega-6, trans fatty acids, and sodium) considered as dietary risk factors by the Global Burden of Disease, the nutritional databases and standardized recipes from the Brazilian Food Composition Table (TBCA) version 7.1 were utilized [23]. This facilitated the decomposition of culinary preparations and processed products, when necessary, into their respective ingredients [24,25].

Adopting a methodology similar to that described by Fulgoni et al. (2018) and Stylianou et al. (2021), the proportional mass of each food group (g) and nutrient (g or mg) per average portion size/g of food items was calculated [15,26]. The GBD separately reports the effects of fibers from fruits, vegetables, legumes, and whole grains, as well as fibers from other sources, given that they demonstrate distinct relationships with the analyzed health outcomes [27]. Consequently, we calculated total fibers as the fraction derived from foods/ingredients not considered fruits, vegetables, legumes, and whole grains to avoid double counting the effects of these latter components [26]. In particular, data regarding omega-6 fatty acids, eicosapentaenoic (EPA), and docosahexaenoic (DHA)—fish omega-3s—in Brazilian foods/ingredients were not available in TBCA 7.1. Thus, values from the U.S. Nutrition Data System for Research (NDSR version 2008) database were borrowed for equivalent items from TBCA 7.1, after adjustments for differences between the national and international sources regarding the total content of polyunsaturated fatty acids (PUFAs) in foods/ingredients [22].

To identify the most consumed food items in Brazil, the contribution of each unique food/beverage to the total daily energy intake was estimated for the general Brazilian population, as well as for the four major regional food systems in the country (explained in Section 2.4), using the approach proposed by Block et al. (1985) [28]. The 20 food items with the highest cumulative individual contributions to total energy intakes were then selected for more detailed HENI analyses. The HENI values were reported per standard portion of food/beverage, which was calculated as the median (P50) of the reported portion size amount for each food item on each consumption occasion, considering the first measurement from the 24-h dietary recall of INA 2017–2018 [16]. All analyses for identifying the energy contribution and portion size of foods were performed with weights, considering the sampling complexity of the survey.

The foods and ingredients consumed by the Brazilian population, according to the INA 2017–2018 [16], were classified according to the Nova classification [19] after being differentiated into recipes and non-recipes according to the Brazilian Food Composition Table (TBCA) [23].

The Nova classification [19] categorizes foods into four groups: in natura and minimally processed, culinary ingredients, processed, and ultra-processed. This stratification of foods was constructed based on the degree of processing of these foods, aiming to classify them according to their culinary use, form of consumption, and health impact, as demonstrated in the Nova classification of foods [19].

In this context, in natura and minimally processed foods are those that undergo no significant modifications, presenting only, as suggested by their definition, minimal processes, represented by fruits, vegetables, legumes, pulses, meats, etc. The second group encompasses culinary ingredients, which are used in food preparation, such as salt, sugar, and oils. Processed foods, in turn, exhibit some degree of processing through the implementation of culinary techniques and the use of culinary ingredients, as seen in cheeses and breads. Lastly, ultra-processed foods achieve a greater degree of processing and are detached from their original food matrix, characterized by the excessive use of culinary ingredients and additives, such as those used to enhance their organoleptic properties [29].

### 2.3. Environmental Impact

To calculate the environmental impacts associated with food production, an adjustment factor was initially applied to each food item reported in the food recalls to account for inedible parts (such as seeds, peels, and bones). Subsequently, the consumed foods were grouped into 53 food categories, such as dairy products, fish, and fruits and vegetables, as described in the WWF Report—Bending the Curve: The Restorative Power of Planet-Based Diets [21]. Next, we multiplied the quantity of raw foods consumed (including peels and seeds) by the environmental impact values presented in the WWF Report, which takes into account Brazilian domestically produced and imported food, encompassing greenhouse gas emissions (kg of CO_2_ equivalents) and water usage (L). This estimate was adjusted by dividing the results by 1000, as the metrics in the WWF Report are based on 1000 g of raw food [21].

### 2.4. Brazilian Food Systems

Foods are analyzed across four food system clusters using the Brazilian Multidimensional Index of Sustainable Food Systems (MISFS-R). This index aims to comprehensively analyze the system through 46 nutritional, social, economic, and environmental indicators across different Brazilian states, identifying four main regional food systems that share similar performance across these indicators, using K-means clustering [20,30]. Foods that contribute more than 0.5% of the total energy consumed by the general population, and specifically within the four clusters, were selected.

The clusters were named based on defining elements. For instance, Cluster A, which includes the states of Acre, Rondônia, Mato Grosso, Mato Grosso do Sul, Goiás, and Tocantins, was designated as the Agricultural Belt due to the strong presence of agricultural and livestock production activities, which exhibit the poorest performance in environmental indicators. Similarly, the other clusters were defined as follows: Cluster B—Central Economic Hub, consisting of Rio Grande do Sul, Santa Catarina, Paraná, São Paulo, Rio de Janeiro, Minas Gerais, and Espírito Santo, characterized by the best overall index and strong performance in the food system domains but also exhibiting the highest rates of inequality based on sex and race in rural areas, as well as increased pesticide use. This cluster is the second highest in terms of reported pesticide poisoning incidents in agriculture; Cluster C—Latent Development Axis, comprising Maranhão, Piauí, Ceará, Rio Grande do Norte, Paraíba, Pernambuco, Bahia, Sergipe, and Alagoas, which scores the lowest on nutritional, income, and productivity indicators; and Cluster D—Vulnerability Frontier, formed by Pará, Amazonas, Roraima, and Amapá, characterized by a scenario of food insecurity, with lower dietary diversity due to reduced access and availability of food. The states within this cluster exhibit a higher burden of chronic childhood malnutrition (Figure S2 in the Supplementary Materials).

### 2.5. Data Analysis

Microsoft Excel^®^ 2021 version spreadsheet software was utilized to conduct descriptive analyses of the data, while the Stata^®^ version 17 and R^®^ version 4.5.0 software packages were employed for the creation of graphical representations to facilitate statistical and exploratory data analysis.

Monte Carlo simulation was used to quantify the uncertainty in DRFs, taking into account uncertainties in dietary intakes, disease burden, and RR in the final estimates. For each age–sex–exposure–risk–outcome combination, we performed 500 draws from the sampling distributions of RR and the uncertainty distributions of dietary intakes, disease burdens, and population sizes (assumed to be normal). These draws were used to generate 500 iterations with estimates of interest for each age–sex group, from which we report the 2.5 and 97.5 percentiles (95% uncertainty interval). These analyses were conducted using SAS software, version 9.4.

In addition, the overall findings for the entire country were analyzed based on their nutritional and environmental impact, as well as by food processing level.

## 3. Results

### 3.1. Brazil

The Brazilian Health Nutritional Index (HENI) was estimated for 1141 foods consumed by the Brazilian population, classifying them in terms of minutes of life lost (negative HENI) or gained (positive HENI) due to disability. Additionally, the environmental impact of these foods was also estimated, resulting in a ranking of foods in each region that contributed more than 0.5% of energy intake (Table S1 in the Supplementary Materials).

When ranked by percentage contribution to energy intake, 33 foods were identified as contributing more than 0.5% to the diets of Brazilians. These foods together accounted for 68.68% of the total energy intake. Among them, unprocessed foods contributed 39.47%, culinary ingredients contributed 6.08%, processed foods contributed 8.64%, ultra-processed foods contributed 8.93%, and culinary preparations contributed 5.56%. These percentages are nested within the total contribution of the 33 foods. Overall, among these 33 foods, the five items that contributed most significantly to energy intake in the country were unprocessed foods, namely rice, beef, beans, chicken, and pork.

Furthermore, seven foods were classified as ultra-processed, accounting for 13.00% of the average daily energy intake. Ultra-processed foods had an average HENI of −16.61 min, ranging from −39.69 min of life (stuffed cookies) to 0.37 min of life (ready-to-drink chocolate milk). Among the 33 most consumed foods in Brazil (Table 1), those associated with the greatest loss of healthy life minutes were ultra-processed foods and meats, such as stuffed cookies (−39.69 min); pork (−36.09 min); margarine with or without salt (−24.76 min); beef (−21.86 min); and salty cookies (−19.48 min). The foods with the best health benefits were freshwater fish (17.22 min); banana (8.08 min); beans (6.53 min); and rice with beans (2.11 min).

**Figure 1:**
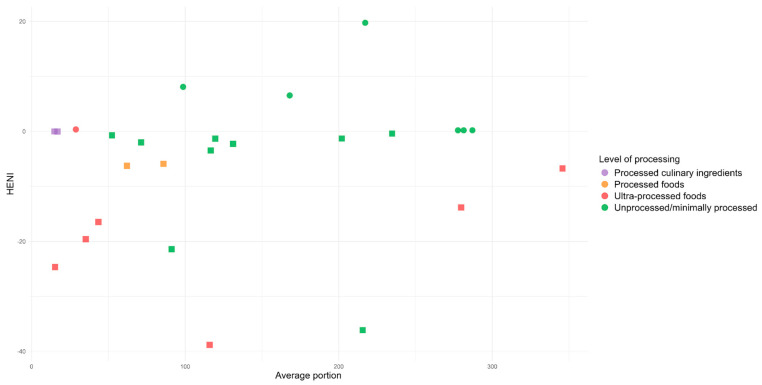
Relationship between the HENI and the most consumed foods in Brazil.

Figure 1 presents the relationship between the HENI and the average portion size of the 33 foods according to the different food groups. Most foods in this ranking—23 in total—showed a negative HENI, whereas only 10 exhibited a positive HENI. In our figures, positive values are represented by circles and negative values are represented by squares. By consulting them in an interactive format (in an HTML version on this journal’s website), it is possible to check the names of the foods represented by each observation in the graphs.

The foods with the best scores mainly belonged to the groups of fish and shellfish, fruits, and vegetables, while those with the worst scores were from the groups of candies and snacks, red meat, and vegetable oils (specifically margarine with or without salt).

When analyzing the environmental impact associated with the studied foods, the greenhouse gas emissions in the country reached up to 21.3 kg CO_2eq_ (beef dish), and water use was as high as 306.1 L (mozzarella pizza). The foods that contributed most negatively to this impact were unprocessed animal-origin foods, such as meats, fish, and dairy products. Conversely, the foods that contributed positively included unprocessed vegetable-origin foods such as beans and vegetable oil and some ultra-processed foods such as salty biscuits and soft drinks.

**Figure 2:**
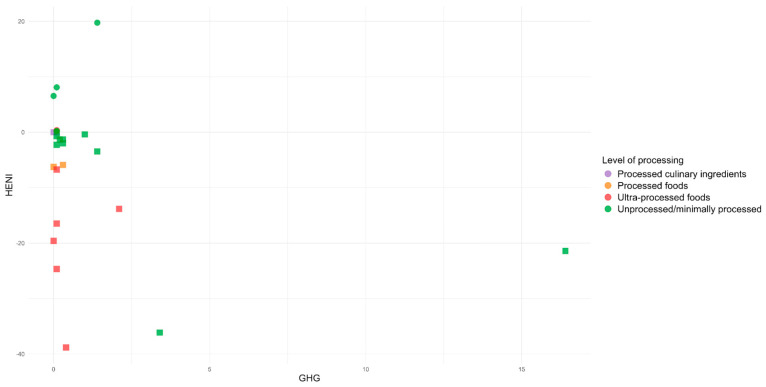
Relationship between the HENI and the environmental impact of foods through greenhouse gas (GHG) emissions in Brazil.

**Figure 3:**
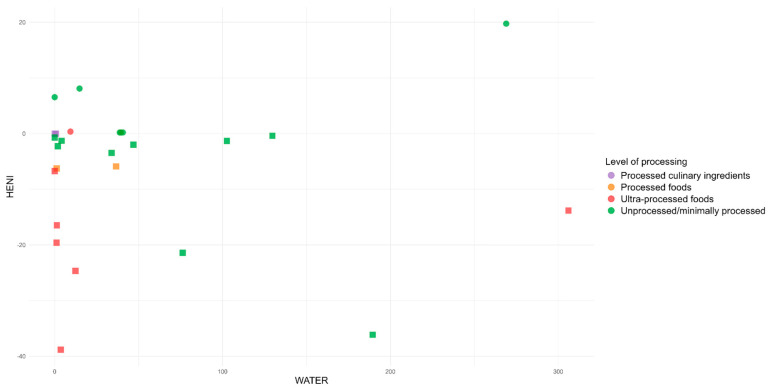
Relationship between the HENI and the environmental impact of foods through water use in Brazil.

From the joint analysis, it was observed that foods with the greatest health benefits and lowest environmental impacts were unprocessed items: banana (8.08 min; 0.1 kg CO_2eq_; 14.8 L) and beans (6.53 min; 0 kg CO_2eq_; 0 L). On the other hand, those that exhibited the least health benefits and the highest environmental impacts were pork (−36.09 min; 3.4 kg CO_2eq_; 189.5 L) and beef (−21.86 min; 16.4 kg CO_2eq_; 76.3 L) (Figure 2 and Figure 3). That is, foods and preparations with ingredients predominantly of animal origin, such as pork, beef, chicken, chicken eggs, and mozzarella pizza, are considered the worst for human health and the environment, while the opposite is true for those of plant origin, such as olive oil and beans, which can be characterized as good food options, regardless of their level of processing (Table 1).

### 3.2. Regional Food Systems

When analyzing the clusters of the Brazilian food systems (as described in Section 2.4 of this study’s methodology) and comparing them to the overall national ranking, little difference was observed in food portion size, as the 52 foods were common across all clusters, such as rice, beans, and beef. However, regional variations in food portion size were still noted, with some foods appearing in only one cluster, such as açaí with granola and cheese bread, or in two clusters, such as dried meat and tapioca. The presence of each food item in Brazil as a whole and in each individual cluster is indicated by an “X” in the spreadsheet; therefore, foods without an “X” for a given cluster are not present in that specific group (Table 2).

**Figure 4:**
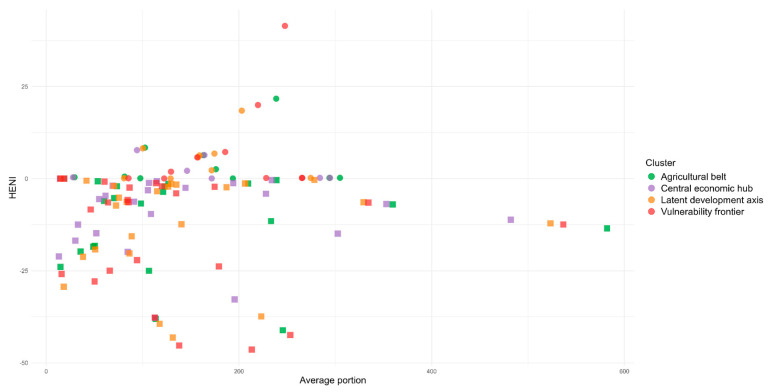
Distribution of the HENI by clusters of Brazilian food systems.

Similar to what was observed for the country, when analyzing the set of clusters of food systems (Figure 4), a predominance of negative HENI scores for the most consumed foods was noted. Among the positive values, foods that belong to the same groups across more than one cluster were identified, such as mixed dishes, fish and shellfish, fruits, and vegetables. Likewise, among the negative values, the red meat group was the most predominant across the four clusters. Additionally, ultra-processed foods frequently appeared on the lists of the most consumed items in all clusters, with them being more prevalent in the Agricultural Belt and the Central Economic Hub, such as margarine (with or without salt), salty biscuits, filled and sweet biscuits, soft drinks, and mozzarella pizza, which all exhibited negative average HENI scores.

**Figure 5:**
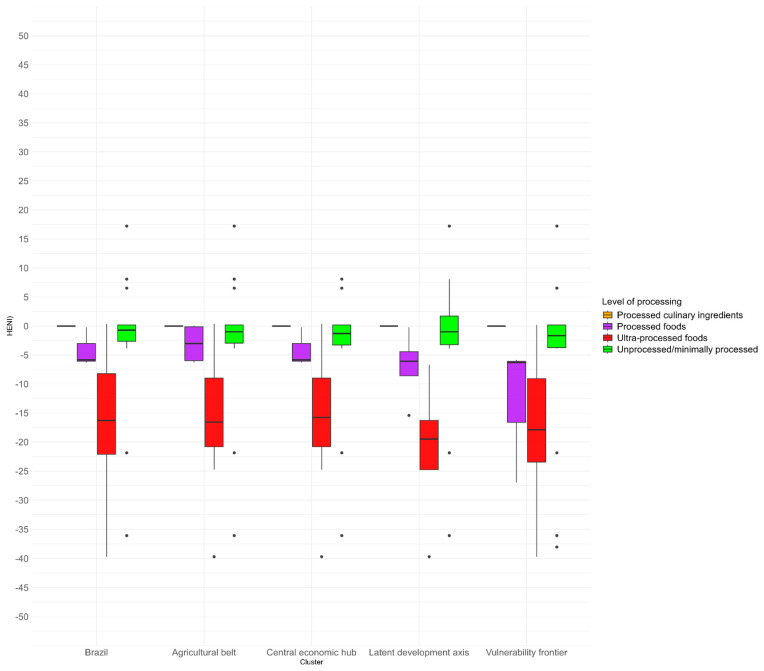
Boxplots of the HENI by clusters of Brazilian food systems, according to the level of processing.

When the HENI results were analyzed according to the food system clusters, based on the level of processing (Figure 5), it was found that the mean index was negative mainly for the consumption of ultra-processed foods in Brazil and in all clusters; however, it showed high variability, with some foods presenting positive HENI values. The average for each cluster was as follows: −16.3’ (Brazil), −16.6’ (Agricultural Belt), −15.7’ (Central Economic Hub), −19.5 (Latent Development Axis), and −17.9 (Vulnerability Frontier). In the Latent Development Axis and Vulnerability Frontier clusters, those with the highest average for ultra-processed foods, the HENI ranged from −61.15’ (dried meat) to 17.22’ (freshwater fish) and from −61.15’ (dried meat) to 41.43’ (açaí with granola), respectively.

**Figure 6:**
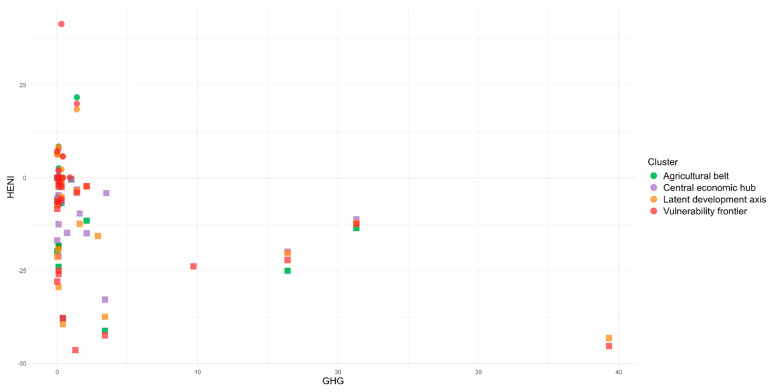
Relationship between the HENI and the environmental impact of foods through greenhouse gas (GHG) emissions in Brazilian regional clusters.

**Figure 7:**
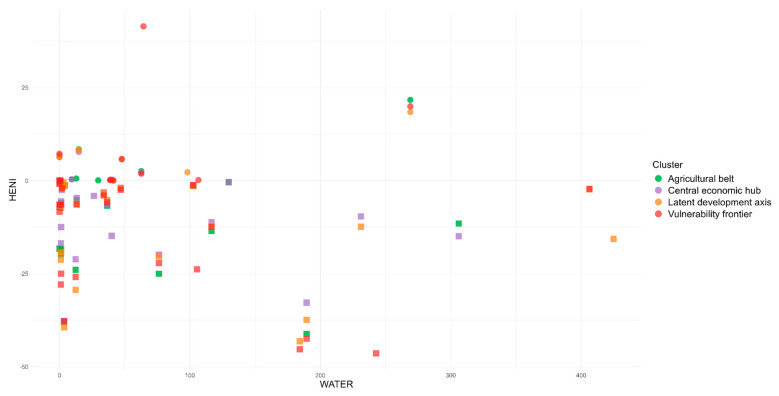
Relationship between the HENI and the environmental impact of foods through water use in Brazilian regional clusters.

In terms of environmental impact, the foods with the greatest impact in terms of GHG emissions and water use in the Agricultural Belt cluster were beef (16.40 CO_2_eq) and meat-based foods (beef dish) (21.30 CO_2_eq) and mozzarella pizza (306.10 L) and freshwater fish (269.00 L), respectively. Similarly, for the Central Economic Hub cluster, the results were as follows: beef (16.40 CO_2_eq) and meat-based foods (such as beef dish) (21.30 CO_2_eq) and mozzarella pizza (306.10 L) and bread with cheese (231.10 L). For the Latent Development Axis cluster, similarly for GHG emissions, the foods with the greatest impact were beef (16.40 CO_2_eq), beef dish (21.3 CO_2_eq), and dried meat (39.30 CO_2_eq), while for water use, they were freshwater fish (269.00 L), whole salt sea fish (406.30), and curd cheese (424.80 L). The same foods from this cluster were found for the Vulnerability Frontier cluster in terms of GHG emissions, and, in relation to water use, whole freshwater fish (242.80 L), freshwater fish (269.00 L) and whole salt sea fish (406.30 L) had the greatest impact (Figure 6 and Figure 7).

## 4. Discussion

In this study, we estimated the Brazilian Health Nutritional Index (HENI) for 1141 foods consumed in Brazil, classifying them by the number of minutes of life gained or lost due to disability and by their environmental impact. Thirty-three foods contributing over 0.5% of dietary energy were identified, with an average HENI of −5.89 min, ranging from −39.69 min (stuffed cookies) to 17.22 min (freshwater fish). The environmental impact of these foods was also assessed, revealing that animal-based foods, such as meats, were associated with higher CO_2_ emissions and water use, whereas plant-based foods, such as bananas and beans, were found to be more beneficial for health and the environment. Fish were an exception among meats, with a highly positive HENI, while pork showed a low negative HENI with reduced carbon impact. Across the Brazilian food systems clusters, it was observed that only around five food groups represent more than 5% of energy contribution, and little variation was observed in food portion size, with predominantly negative HENI values for animal-based foods like meats, and ultra-processed foods like cookies, while plant-based foods and minimally processed foods, such as fruits and fish, showed positive values.

Food biodiversity is characterized by the variety of plant, animal, and other species used for food within a local, regional, or national ecosystem [31]. Conversely, dietary monotony refers to a pattern in which an individual or population consumes a limited range of foods, leading to a lack of variety that can result in nutritional deficiencies [32]. Dietary monotony can be observed, for instance, in Mendonça et al. (2019), who studied the intake of fruits and vegetables in Belo Horizonte, Minas Gerais—a state in Southeast Brazil. While consumption levels were adequate, they lacked diversity and were influenced by the consumer’s environmental conditions [32,33]. Household availability of foods from Brazil’s biodiversity, such as açaí and dried meat, is essential for promoting dietary diversity. However, as noted by Gomes (2023), although Brazil holds about 20% of the planet’s biodiversity and some of the world’s richest biomes, the availability of foods derived from this biodiversity remains limited [31]. Additionally, the observed HENI results showed that freshwater fish (17–22 min), banana (8.08 min), beans (6.53 min), and rice and beans (2–11 min) were positively associated with health, highlighting the importance of biodiversity in promoting healthier diets, especially among the younger generation within regional food systems. These findings could be valuable when recommending foods to be prioritized in public health strategies aimed at improving dietary diversity and health outcomes across Brazil.

Brazil is rich in biodiversity, boasting a wide range of fruits, vegetables, legumes, and meats. Studies emphasize the importance of considering the health and environmental impacts of both plant- and animal-based foods. Plant-based foods, such as beans and olive oil, show positive health impacts, while red meats are linked to negative health and environmental consequences, showing in natura products can have different health and environmental impacts [34,35]. This distinction should be incorporated into the population’s food practices. However, the frequency of minimally processed food consumption remains limited, with only 30.9% of the population consuming five or more groups of protective foods the day before the interview [36]. In environmental terms, agribusiness is responsible for 70.45% of the country’s water use, highlighting the urgent need to rethink food production and consumption models, especially regarding beef, which is the most resource-intensive food associated with minutes lost to disability [37,38]. This analysis of food consumption reflects not only the environmental and health impacts but also reveals additional challenges related to non-communicable diseases (NCDs) in Brazil.

Large-scale agriculture is predominantly oriented toward export products in the country, such as soy, sugarcane, and corn—referred to as commodities. It is estimated that around 70% of the country’s agricultural production is destined for foreign markets, with soy being the leading product, accounting for over 50% of agricultural exports, much of which is directed to China [39,40]. This production model is mainly concentrated in the central–west and parts of the south, where industrial agriculture and large landholdings are prevalent. Land concentration in these regions has sparked debates about unequal land access and its environmental consequences [41,42].

On the other hand, family farming accounts for much of the food consumed domestically and is more diverse and geographically dispersed [43]. These small-scale producers are present throughout the country, with a stronger presence in the northeast and south regions. The production of foods such as beans, cassava, fruits, and vegetables is closely linked to family farming, which, despite occupying a smaller share of land compared to agribusiness, plays a fundamental role in supplying food for domestic consumption and promoting food security, particularly in rural and vulnerable communities [37,44]. This contrast between family farming and agribusiness highlights one of the challenges faced in promoting healthy and sustainable diets, especially considering how the increase in consumption of ultra-processed foods delays efforts and further hinders the achievement of food security and diet quality among the population.

Studies by Martins et al. (2021) [45] and Levy et al. (2023) [9] showed that increased consumption of ultra-processed foods is associated with poorer diet quality and increased social vulnerabilities. Difficulties in accessing fresh and minimally processed foods, as highlighted by Levy et al. (2023), obscure critical concepts related to a balanced diet, such as the origin and environmental repercussions of food choices, exacerbated by the increase in advertising of these ultra-processed foods that influence consumers’ personal choices [9]. This conceptual opacity prevents the promotion of healthy eating habits, which is in line with the Human Right to Adequate and Healthy Food, enshrined in the Federal Constitution [46], and the Sustainable Development Goals (SDGs) [47].

The prevalence of non-communicable diseases (NCDs) in Brazil is highest in the south and southeast regions. The National Plan for the Combat of Non-Communicable Diseases (2021–2024) aims to reduce mortality through specific actions, such as increasing fruit and vegetable consumption [48]. This is part of a broader effort that was renewed and expanded with the adoption of the “Global Strategy for the Prevention and Control of Noncommunicable Diseases 2013–2030” by the World Health Organization, which seeks to reduce premature mortality from NCDs by 25% by 2025, using 2010 as the baseline [49]. Moreover, the implementation of public health measures and policies is also aligned with the SDGs, which include targets such as promoting healthy diets and ensuring access to nutritious food for all [47]. This is particularly relevant for reducing premature mortality caused by NCDs, with Brazil being a member of the WHO and directly committed to following these guidelines, reflecting a continuity in government actions in recent years [36]. Additionally, dietary patterns observed in other regions of the country may also be associated with the prevalence of NCDs and other health outcomes, highlighting the need for region-specific strategies that consider cultural, economic, and environmental differences in food consumption.

### 4.1. Recommendations for Future Research

The study by Stylianou et al. (2021), in which the authors developed the HENI, showed that small dietary changes focused on specific foods can provide substantial benefits for both human health and environmental sustainability [15]. Their approach, which evaluates the simultaneous impacts of various food choices, is similar to the method in this study by comparing the benefits of minimally processed and plant-based foods with the costs of ultra-processed options. Both studies emphasize that adopting a diet richer in plant-based foods can significantly reduce greenhouse gas emissions and mitigate negative health impacts. In our study, the HENI values varied from −39.69 min of healthy life (for stuffed cookies) to 17.22 min (for freshwater fish), while Stylianou et al. [15] reported a range from −71 min (corned beef with tomato sauce and onion) to 82 min gained (sardines with a tomato-based sauce). In line with Stylianou et al. (2021) [15], the HENI we used adopts the Nova classification of foods—according to their level of processing—and is also adaptable, as discussed by the authors, as new epidemiological data, nutritional specificities, and public health nutrients, such as vitamin D and potassium, are incorporated [28,34]. This continuous adaptation of the HENI would allow for adjustments to evolving dietary recommendations, improving its precision and applicability. Furthermore, Stylianou et al. (2021) [15] emphasize the potential for personalized diets, enabling consumers to make substitutions that reduce environmental impacts and improve health, such as replacing processed meats with fish. This kind of model can inform food policies, government guidelines, educational programs, and labeling campaigns, fostering healthier and more sustainable food choices. With this flexibility, our HENI and similar approaches can, therefore, guide food policy and adjustments that consider nutritional benefits, encouraging a gradual transition toward diets that promote health and environmental sustainability. Our findings provide valuable insights into the real-world consequences of individual and institutional food choices, demonstrating their measurable impacts on health and the environment. By moving beyond theoretical assumptions, this evidence strengthens the case for integrating sustainability into public food policies, including dietary guidelines that consider regional specificities and environmental concerns alongside nutritional recommendations.

This study covers 1141 foods consumed in Brazil based on a recognized and consolidated national database, offering a robust overview of dietary habits across different regions and food systems in the country. Additionally, the approach allowed for a detailed analysis of the health and environmental impacts of each food by integrating health and environmental effects through the combination of HENI data with greenhouse gas emissions and water usage. This provided insights into the consequences of food choices, moving beyond theoretical assumptions, and highlighting the relevance for both public health and environmental sustainability. Such insights are particularly important given the growing role of food in discussions about climate change, plant-based and ultra-processed foods, and biodiversity.

### 4.2. Limitations

However, while the study explored regional variations, it did not analyze barriers related to access, acquisition, or other factors affecting the consumption of fresh and minimally processed foods, nor did it address solutions to increase dietary diversity across regions in the country.

Although the proposed nutritional index for the most commonly consumed foods in Brazil’s regional food systems is an innovative tool, it may present several limitations. One of these concerns is the availability and quality of data on food consumption across different regions, which may be incomplete or outdated, affecting the accuracy of the index. Furthermore, Brazil’s considerable cultural and regional diversity can make it difficult to capture dietary variations, especially when the index is based solely on the most consumed foods. Another challenge is integrating environmental and health impacts into a single index, as these dimensions involve complex interactions that may not be fully represented in a simplified model. Socioeconomic and demographic factors, such as income and education levels, can also influence food choices and, consequently, health outcomes, which may be difficult to incorporate comprehensively into the index. Additional limitations include shifts in dietary patterns over time, such as the increase in the consumption of ultra-processed foods, which require constant updates to maintain the tool’s relevance.

In addition, it is important to note the absence of data on sugar use as a risk factor, which is not covered in Global Burden of Disease (GBD) analyses. This omission not only limits the understanding of the risks associated with excessive sugar consumption but also hampers the development of effective public policies to promote healthy and sustainable diets in Brazil. Similarly, the term “beef dish” was used to group preparations that contain both meat and vegetables, providing a practical approach for nutritional analysis. This term allows for a simplified and comparative evaluation of dishes that, due to their composition, can vary in the concentration of meat and vegetables. The nutritional index associated with “beef dish” can be elevated due to two main factors: the concentration of meat in the dish, which tends to increase the overall weight of the preparation, and the associated environmental impacts, such as the high use of water and the emission of greenhouse gases, particularly carbon dioxide (CO_2_). Thus, the amount of meat not only directly influences the nutritional profile of the dish but also significantly impacts its environmental footprint, raising the index value due to both the nutritional load and the environmental costs associated with meat production.

Furthermore, the extrapolation to health effects is in fact influenced by multiple factors, including individual dietary patterns, lifestyle, and genetic predispositions, which are not fully captured in our analysis.

Moreover, it is important to highlight that the results of the index are based on estimates, as we are analyzing individual foods rather than the diet as a whole. When comparing foods by their level of processing, we observe that both unprocessed and minimally processed foods exhibit both positive and negative nutritional indices. While, on average, ultra-processed foods tend to show negative results and unprocessed foods vary between positive and negative, this could suggest that the analysis may be biased. This approach is essentially useful for a more in-depth investigation of the impacts of the burden of disease attributed to the diet of Brazilians and its impacts in terms of years of life lost and mortality and may be more useful when applied to specific food groups, such as meats, dairy products, etc., allowing for a more detailed and accurate analysis. Finally, the acceptance and implementation of the index as a public policy tool may be challenging due to cultural resistance and the need for adjustments to existing food policies. These limitations can be addressed through a continuous process of data collection and analysis, along with interdisciplinary collaboration to ensure the index’s effectiveness and applicability. A relevant example is the Nutri-Score, a front-of-pack labeling system recommended by the European Union to encourage healthier food choices. Similar initiatives demonstrate how structured approaches to food classification can support public health efforts, reinforcing the importance of scientifically validated indices in shaping effective food policies [50].

## 5. Conclusions

The analysis of the foods most commonly consumed in Brazil and their impacts on both the environment and health enables more informed dietary choices. It highlights foods with better rankings in terms of both the gain and loss of minutes of life, as well as their environmental footprints. The results revealed that the most consumed foods in the country, such as rice, beans, and meats, have lower processing levels but show significant variations in their environmental footprints and health impacts. These findings emphasize the need for a more nuanced understanding of dietary patterns and their broader implications for public health and sustainability.

Given these insights, it is crucial to promote a culture of consumption that favors environmentally friendly and healthy products. Public policies aimed at improving access to and supply of healthy foods are essential to mitigate the effects of social inequality on consumption. Specific strategies should include promoting dietary shifts away from some foods, such as cookies and pizzas, and red meats, which are associated with significant environmental and nutritional impacts, according to our study. These changes can be supported through incentive systems, regulations, and policies that encourage households to reduce consumption of products harmful to both human health and the environment.

Our study advances the field by providing a novel adaptation of the HENI methodology to the Brazilian context, offering a comprehensive tool to assess both the health and environmental impacts of dietary patterns in different parts of the country. The findings underscore the urgency of integrating these metrics into public health and environmental policies. Future research should focus on refining the methodology to capture municipality-specific dietary characteristics and exploring the long-term effects of dietary changes on public health and sustainability. Additionally, individual and household-level research is necessary to better understand the factors driving consumption patterns and to develop more targeted interventions for healthier, more sustainable diets.

## Figures and Tables

**Table 1:** Foods contributing more than 0.5% of the total energy consumed by the Brazilian population, classified according to the Nova classification, along with their respective HENI values, carbon dioxide emissions (in kg CO_2eq_), water use (in liters), and average consumption (in grams).

Nova Classification	Food	Caloric Contribution (kcal)	Average Portion Size (g)	HENI (‘)	GHG (kg CO_2eq_)	Water Usage (L)
In natura or minimally processed	Rice	10.36	119.63	−1.30	0.20	102.60
	Beef	6.82	91.17	−21.86	16.40	76.30
	Beans	6.43	168.07	6.53	0.00	0.00
	Chicken	5.55	116.69	−3.88	1.40	33.90
	Pork	2.43	215.59	−36.09	3.40	189.50
	Pasta	1.54	131.18	−3.27	0.10	1.90
	Chicken eggs	1.05	71.29	−2.00	0.30	46.90
	Cassava flour	1.00	52.35	−0.70	0.10	0.00
	Banana	0.93	98.66	8.08	0.10	14.80
	Cassava	0.73	202.01	−1.32	0.30	4.10
	Freshwater fish	0.56	217.23	17.22	1.40	269.00
	Juice	0.56	277.62	0.19	0.10	38.80
	Natural fruit juice	0.51	281.36	0.20	0.10	39.40
	Orange juice	0.50	287.03	0.20	0.10	40.60
	Whole milk	0.50	234.75	−0.40	1.00	129.70
Culinary ingredient	Sugar	4.62	16.89	−0.02	0.10	0.80
	Olive oil	1.46	14.86	0.00	0.00	0.00
Processed	French bread	7.02	62.08	−6.28	0.00	1.20
	Wheat cake	0.83	85.83	−5.85	0.30	36.60
	Beer	0.79	1178.32	−0.23	1.30	11.50
Ultra-processed	Margarine with or without salt	2.59	15.29	−24.76	0.10	12.40
	Salty cookies	1.62	35.28	−19.48	0.00	1.10
	Soft drinks	1.37	345.70	−6.67	0.10	0.00
	Sweet biscuits	1.04	43.47	−16.26	0.10	1.30
	Stuffed cookies	0.90	115.98	−39.69	0.40	3.60
	Ready-to-drink chocolate milk	0.73	28.83	0.37	0.10	9.40
	Mozzarella pizza	0.68	279.75	−9.76	2.10	306.10
Culinary preparations	Coffee with milk	1.56	153.71	0.05	0.40	41.30
	Rice with beans	1.00	145.38	2.11	0.10	62.70
	Beef dish ^1^	0.94	516.79	−11.27	21.30	116.60
	Bread with butter	0.81	72.89	−5.50	0.10	13.20
	Couscous	0.66	131.97	−1.60	0.10	2.60
	Bread with cheese	0.59	125.48	−11.09	1.60	231.10

^1^ Included preparations with red meat and vegetables.

**Table 2:** Presence of foods in Brazil and regional clusters, according to a caloric contribution ranking equal to or greater than 0.5%.

Food	Brazil	Cluster			
		A—Agricultural Belt	B—Central Economic Hub	C—Latent Development Axis	D—Vulnerability Frontier
Sugar	X	X	X	X	X
Rice	X	X	X	X	X
Sweet biscuits	X	X	X	X	X
Stuffed cookies	X	X	X	X	X
Salty cookies	X	X	X	X	X
Wheat cake	X	X	X	X	X
Coffee with milk	X	X	X	X	X
Beef	X	X	X	X	X
Pork	X	X	X	X	X
Beans	X	X	X	X	X
Chicken	X	X	X	X	X
Pasta	X	X	X	X	X
Margarine with or without salt	X	X	X	X	X
Chicken eggs	X	X	X	X	X
Bread with butter	X	X	X	X	X
French bread	X	X	X	X	X
Soft drinks	X	X	X	X	X
Beef dish	X	X	X	X	X
Rice with beans	X	X	X		X
Olive oil	X	X	X		X
Banana	X	X	X	X	
Beer	X	X	X	X	
Cassava flour	X	X		X	X
Cassava	X	X	X	X	
Freshwater fish	X	X		X	X
Natural fruit juice	X	X		X	X
Whole milk	X	X	X		
Bread with cheese	X		X	X	
Mozzarella pizza	X	X	X		
Juice	X		X		X
Orange juice	X	X	X		
Ready-to-drink chocolate milk	X	X			
Dried meat				X	X
Bread with egg				X	X
Whole salt sea fish				X	X
Tapioca				X	X
Açaí with granola					X
Baião de dois				X	
Sweet potato				X	
English potato			X		
Fuba cake		X			
Barbecue					X
Couscous				X	
Farofa					X
Rope beans				X	
Sausage			X		
Noodle with meat			X		
Porridge					X
Cheese bread		X			
Whole freshwater fish					X
Coalho cheese				X	
Lettuce, tomato, and onion salad		X			

## Data Availability

The data used in this study are derived from public domain resources and are openly available through the following sources: IBGE’s website: https://www.ibge.gov.br/pesquisa-de-orcamentos-familiares, accessed on 13 March 2023. Global Burden of Disease, 2019: https://www.healthdata.org/research-analysis/gbd, accessed on 22 April 2023. World Wildlife Fund, 2020, available on the publications website: https://www.worldwildlife.org/publications/bending-the-curve-the-restorative-power-of-planet-based-diets, accessed on 9 June 2023.

## Supplementary Materials

The following supporting information can be downloaded using the following link: https://www.mdpi.com/article/10.3390/ijerph22050745/s1. Figure S1: Schematic diagram of the process followed to characterize the environmental and health impacts of the life cycle of individual food items: The diagram illustrates an average portion size of rice as an example (Stylianou et al., 2021 [15]); Figure S2: Clusters of Brazilian food systems (adapted from Norde et al., 2022 [20]); Table S1: Foods consumed in Brazil and in food systems (clusters A to D) with caloric contribution above 5% and their respective Health Nutritional Index (‘), greenhouse gas emissions (kg CO_2_ equivalents), and water use (liters).
