# Supplementary material for: Health and Environmental Impacts of Major Foods Consumed in Regional Food Systems of Brazil
Source: Int J Environ Res Public Health. 2025 May 9;22(5):745. doi: 10.3390/ijerph22050745 (PMC12111131; doi:10.3390/ijerph22050745)
Supplement: Supplementary file 1 [file ijerph-22-00745-s001.zip › Supplementary Figure S1. Schematic diagram of process followed to characterize the health and environmental impacts of individual food items Diagram illustrates an average portion size of rice as an example (adapted from Stylianou et al, 2021).pdf]

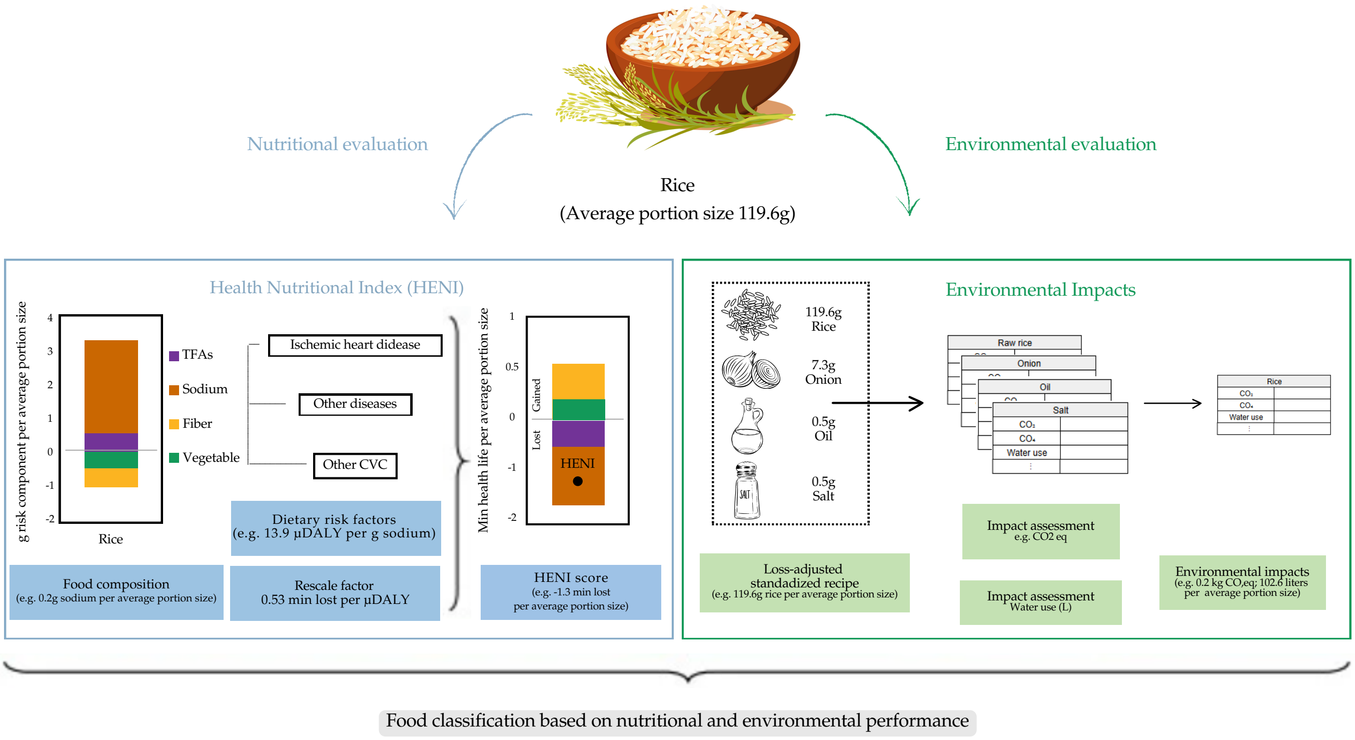
Nutritional evaluation
Environmental evaluation
Rice
(Average portion size 119.6g)
Health Nutritional Index (HENI)
g risk component per average portion size
4
3
2
1
0
-1
-2
Rice
TFAs
Sodium
Fiber
Vegetable
Ischemic heart didease
Other diseases
Other CVC
Dietary risk factors
(e.g. 13.9 µDALY per g sodium)
Food composition
(e.g. 0.2g sodium per average portion size)
Rescale factor
0.53 min lost per µDALY
Min health life per average portion size
1
0.5
0
-1
-2
Gained
Lost
HENI
HENI score
(e.g. -1.3 min lost
per average portion size)
Environmental Impacts
119.6g
Rice
7.3g
Onion
0.5g
Oil
0.5g
Salt
Raw rice
Onion
Oil
Salt
Water use
Rice
Water use
Impact assessment
e.g. CO2 eq
Impact assessment
Water use (L)
Loss-adjusted
standadized recipe
(e.g. 119.6g rice per average portion size)
Environmental impacts
per average portion size)
Food classification based on nutritional and environmental performance
