# Supplementary figures and images for: Health and Environmental Impacts of Major Foods Consumed in Regional Food Systems of Brazil
Source: Int J Environ Res Public Health. 2025 May 9;22(5):745. doi: 10.3390/ijerph22050745 (PMC12111131; doi:10.3390/ijerph22050745)

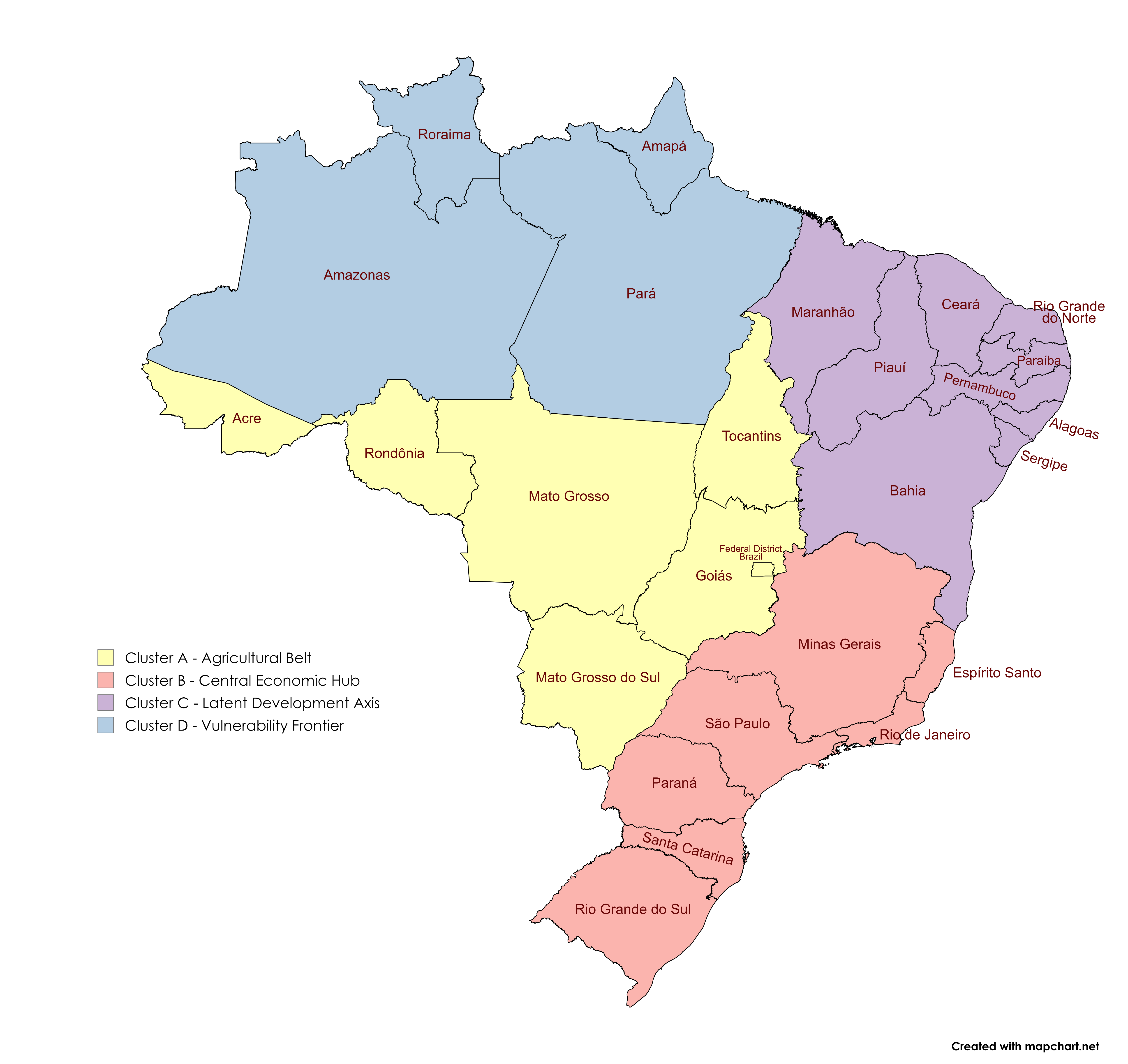

Supplement: Supplementary file 1 [file ijerph-22-00745-s001.zip › Supplementary Figure S2. Clusters of Brazilian food systems (Norde et al., 2022).png]
